# Detection of macrotrabecular-massive hepatocellular carcinoma based on viscoelastic characteristics obtained by multifrequency magnetic-resonance elastography

**DOI:** 10.1007/s00330-025-12024-y

**Published:** 2025-10-09

**Authors:** Zhen Wang, Qi Liang, Jia Luo, Yunjie Liao, Junhong Duan, Qin Liu, Qiyuan Chen, Ze Mi, Hongpei Tan, Pengfei Rong

**Affiliations:** 1 Department of Radiology, The Third Xiangya Hospital of Central South University, Changsha, China; 2 Department of Hepatobiliary and Intestinal Surgery, Hunan Cancer Hospital, Changsha, China

**Keywords:** Hepatocellular carcinoma, Elasticity imaging techniques, Magnetic resonance, Subtype

## Abstract

**Objectives:**

To investigate the use of viscoelastic characteristics obtained with magnetic-resonance elastography (MRE) in identifying the macrotrabecular-massive (MTM) subtype of hepatocellular carcinoma (HCC) and its association with gene expression profiles.

**Materials and methods:**

Fifty-one patients (mean age, 56.2 ± 12.6 years; 42 men) with histologically proven HCCs (16 with the MTM subtype, and 35 without) and 47 healthy participants (mean age, 54.1 ± 13.7 years, 24 men) underwent preoperative MRI and MRE examinations and were prospectively enrolled. Tumor viscoelasticity (comprising *c* and *φ*), imaging features and clinical information were analyzed and diagnostic models developed. Logistic regression and area-under-the-curve (AUC) methodology evaluated the models’ efficacy for determining the MTM-HCC. RNA sequencing and KEGG pathway analyses identified differential gene expression between 12 high-*c* and 12 low-*c* tumor samples.

**Results:**

In HCC patients with elevated Edmondson–Steiner grades, satellite nodules, non-smooth margins, fat deficiency, or an arterial phase hypovascular component (APHC) more than 20%, tumor viscoelastic values *c* or *φ* were higher, compared with patients without these features (*p* < 0.05). Tumor *c* (T-*c*) was an independent predictor of MTM-HCC (AUC, 0.818; 95% confidence interval: 0.685, 0.950; *p* < 0.001); Combining T-*c* with ≥ 20% APHC yielded a higher AUC (0.843), but not significantly different from T-*c* alone (*p* = 0.533). RNA sequencing showed high-*c* tumors upregulated cell proliferation and DNA replication genes but downregulated immune regulation genes.

**Conclusion:**

MRE-derived T-*c* is a promising non-invasive biomarker for identifying MTM-HCC. HCCs with different T-*c* levels show distinct gene expression profiles, particularly in proliferation and immune pathways. Research with larger cohorts is needed to validate clinical utility.

**Key Points:**

***Question*** *Can MRE-based viscoelastic values identify the macrotrabecular-massive (MTM) subtype of HCC?*

***Findings*** *Tumor-c based on MRE has a unique diagnostic performance for identifying MTM-HCC; tumor stiffness correlates with proliferative and immune gene expression.*

***Clinical relevance*** *MRE-based stiffness is a noninvasive predictor of MTM-HCC, and high-stiffness tumors show upregulation of proliferation genes and downregulation of immune genes. These findings may guide personalized treatment, but larger studies are required to confirm clinical applicability.*

**Graphical Abstract:**

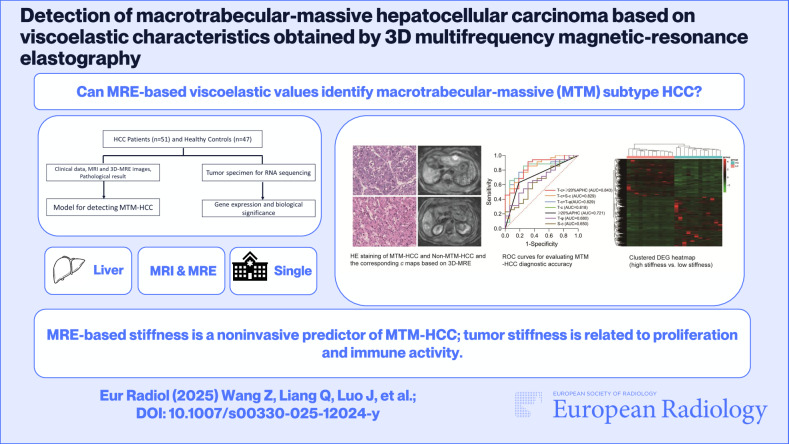

## Introduction

Hepatocellular carcinoma (HCC) is the most common type of primary liver cancer, accounting for over 80% of cases [1]. Despite advances in diagnostic and therapeutic approaches, the prognosis for HCC patients remains poor, with significant variability in clinical outcomes. The classification of HCC based on its molecular, imaging, and pathological features plays a crucial role in determining treatment strategies [2]. Among the different subtypes, the macrotrabecular-massive (MTM) subtype stands out for its highly aggressive nature and poor prognosis [3, 4]. However, the identification of this subtype requires pathological histological validation, and it would be beneficial to develop a more precise treatment strategy if this subtype can be predicted using noninvasive imaging biomarkers before surgery.

Magnetic resonance elastography (MRE) is a noninvasive imaging technique that enables the assessment of liver stiffness [5, 6], a key parameter closely linked to treatment outcomes and prognosis in liver-related diseases [7, 8]. Evidence increasingly supports the role of MRE in the diagnosis, treatment planning, and prognostication of HCC [9–13], with further applications in predicting response to local therapies and immunotherapy [14–16]. However, the potential of MRE to distinguish the MTM-HCC subtype remains unexplored. Moreover, while MRE-derived parameters reflect tumor mechanical properties, the underlying biological mechanisms, including their relation to tumor gene expression profiles, are incompletely characterized. To our knowledge, accumulating evidence demonstrates the association between tumor mechanical properties and malignancy [17–19], highlighting the need to develop mechanical imaging biomarkers for identifying aggressive tumor subtypes and advancing clinical translation.

In this study, we integrate MRE as a noninvasive imaging modality for preoperative prediction of the MTM-HCC subtype. To explore the gene expression of the observed mechanical properties, we also perform RNA sequencing on tumor tissue specimens. Notably, RNA sequencing serves as a complementary research tool for understanding tumor biology and validating imaging biomarkers rather than a direct preoperative diagnostic method, as it requires invasive tissue sampling.

## Materials and methods

### Participants

This prospective single-center cohort study was performed at the Third Xiangya Hospital of Central South University between January 2023 and December 2024 with institutional review board approval (Identification: Fast I 22269); consecutive participants gave written informed consent for MRE examination and tumor tissue RNA sequencing, as well as related pathologic validation. Patients with clinically suspected hepatocellular carcinoma who were willing to undergo surgical treatment and aged 18 or above were included. The exclusion criteria included (1) preoperative MRE examination was not completed, or the image quality did not meet the standards; (2) history of liver transplantation, local or regional treatments, or chemotherapy before surgery; (3) no surgery was performed during hospitalization; or (4) the pathological findings were benign or indicated other malignant tumors of the liver. We also recruited healthy controls who had no prior history of major diseases or chronic diseases and had negative MRI results. The criteria for exclusion of healthy controls included (1) abnormal findings on T2-weighted image (T2WI) and on diffusion-weighted images (DWI) or (2) significant liver fat deposition observed on the in-phase and opposed-phase imaging.

### Conventional MRI and MRE

Conventional MRI examinations were performed with a 3-T scanner equipped with a 32-channel phased-array surface coil. The liver imaging protocol included T1-weighted images (T1WI), T2WI, DWI with b values of 0, 50 and 800 s/mm^2^, and dynamic contrast-enhanced (DCE) imaging. An MRE device is connected to the scanner for MRE. All patients fasted for at least 4 h prior to the examination. The sequence setup is similar to that described by Liu et al [20]. Multifrequency wave field data were processed using specialized software, available at https://bioqic-apps.com, to generate magnitude and parametric maps; shear wave speed (related to *c*) and loss angle (related to *φ*) of the complex shear modulus were obtained as markers of stiffness and viscous properties, respectively. Detailed parameters of the scanning method and inspection sequence are provided in the Supplementary Material.

### Image analysis

Two experienced radiologists (one with 7 years of radiographic experience, and another with 3 years of experience) reviewed all preoperative MRI features in consensus; they were blinded to the details of the patients’ clinical data and pathological results. For each lesion, these radiologists independently evaluated the following imaging features of each HCC: (1) non-smooth margin (Non-SM), (2) shape of tumor, (3) size ≥ 5 cm, (4) arterial phase hypovascular component (APHC) ≥ 20% [21], (5) non-rim arterial phase hyperenhancement (APHE), (6) non-peripheral washout, (7) enhancing capsule, (8) non-enhancing “capsule,” (9) nodule in nodule, (10) mosaic architecture, (11) intratumoral hemorrhage (IH), (12) intratumoral fat (IF), (13) arterial phase peritumoral enhancement, (14) substantial intratumoral necrosis (> 20%), (15) intratumoral artery (IA), (16) central enhancement at the delayed phase [21, 22], and (17) ascites. The definitions of MRI features are shown in Supplementary Table S1.

The image processing software ImageJ (version 1.54 k) was used to synchronize the magnitude, *c*, and *φ* maps, and to select regions of interest (ROIs) based on the magnitude map. With these ROIs accurately mapped to the aligned *c* and *φ* maps, precisely localized *c* and *φ* values were obtained and considered to represent, respectively, the stiffness and viscosity of the tissue. Each ROI was defined to include only the tumor and liver parenchyma while avoiding the boundaries of the large blood vessels and the necrotic portion of the tumor.

### Histopathological examination

A pathologist with 7 years of experience in liver pathologic analysis, and blinded to all radiological and clinical results, reviewed all surgical specimens. Hepatocellular carcinoma subtypes were recorded and classified according to the World Health Organization (2019) [23]; the MTM-HCC subtype is defined as a predominant (> 50% of the tumor area) macrotrabecular (trabeculae more than six cells thick) architectural pattern at hematoxylin-eosin staining. The remaining hepatocellular carcinomas are defined as non-MTM subtype [24]. Immunohistochemistry was performed to detect the status of Ki67 expression.

### RNA sequencing and analysis

We divided 51 patients into a high-*c* (2.316–3.731 m/s) group and a low-*c* (1.247–2.316 m/s) group based on the median value of T-*c* (2.316 m/s); in each group, 12 tumor samples were obtained. RNA sequencing was performed on the Illumina NovaSeq platform. Differential expression analysis was conducted using the DESeq2 package (version 1.34.0) in the R software ecosystem (version 4.1.0). Gene expression levels were quantified as raw read counts, and differential expression was determined between the high-*c* and low-*c* groups. Genes with an adjusted *p*-value < 0.05 and the | log_2_FoldChange| > 1 were considered significantly differentially expressed. The top 500 upregulated and 500 downregulated genes were selected for further functional enrichment analysis. Kyoto Encyclopedia of Genes and Genomes (KEGG) pathway enrichment analyses were performed using the clusterProfiler R package. KEGG pathways with a *p*-value < 0.05 were considered significantly enriched. For visual representation, bubble plots were generated, displaying the gene ratio, count of associated genes, and significance levels. All statistical analyses were performed using the software R. Differential gene expression was analyzed using the DESeq2 package, and the results were visualized using volcano plots and heatmaps.

### Statistical analysis

Statistical analyses were performed using IBM SPSS Statistics 27; graphs were generated with GraphPad Prism 8. A *p*-value < 0.05 was considered statistically significant. Continuous variables were tested for normality and are shown as medians and IQR or means and standard deviation; categorical variables are shown as frequency and percentage. Differences between groups were analyzed using T-tests or nonparametric tests for continuous variables, and Fisher exact tests for categorical variables. The independent predictors of MTM subtypes were determined by a stepwise selection of univariate regression analysis and multivariate logistic regression analysis; these predictors were used to construct the prediction model. The diagnostic performance of each parameter model for preoperative identification of MTM-HCC was evaluated by the area under the receiver operating characteristic (ROC) curve (AUC), and the AUC values were compared using the Delong test. Interobserver agreement was assessed using Cohen’s kappa or the intraclass correlation coefficient (ICC). The correlation was calculated using the Spearman correlation coefficient.

## Results

### Participant baseline characteristics

**Fig. 1:**
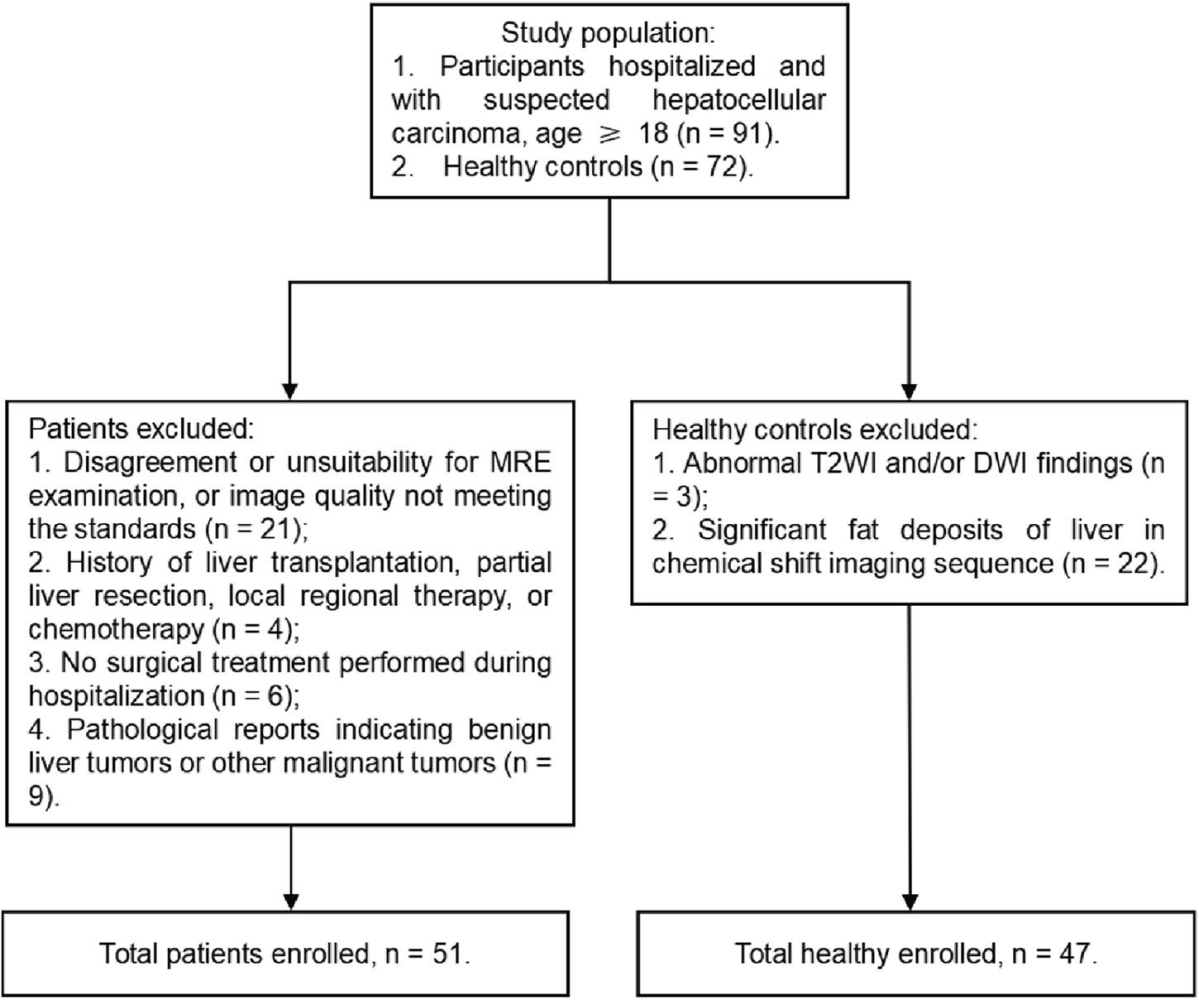
Flowchart of participant inclusions and exclusions

The participant recruitment flowchart is shown in Fig. 1. The study cohort included 51 pathology-confirmed HCC patients (mean age 56.2 ± 12.6 years; range, 30–77 years), comprising 42 (82.4%) males and 9 (17.6%) females. Among them, 42 and 4 patients were infected respectively with hepatitis B virus (HBV) and hepatitis C virus (HCV). Regarding liver function classification, 46 patients (90.2%) were Child–Pugh A and 5 (9.8%) were Child–Pugh B, with no Child–Pugh C cases. Additionally, the study included 47 healthy controls (mean age 54.1 ± 13.7 years, range 25–77 years), consisting of 24 males and 23 females. The characteristics of all enrolled patients (Including Non-MTM-HCC subtype categories) and healthy controls are summarized in Supplementary Table S2.

### HCC characteristics and viscoelastic parameters

**Fig. 2:**
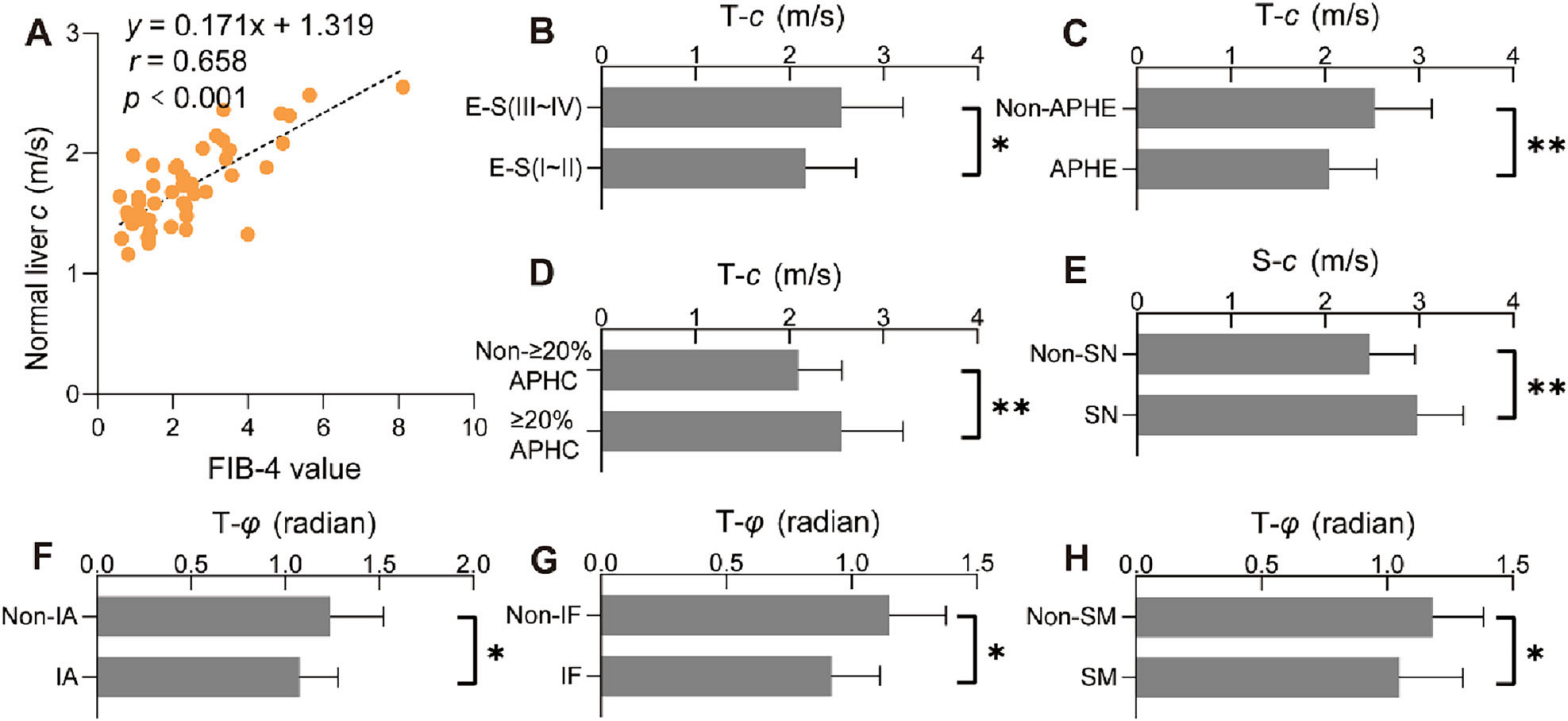
**A** Correlation between the *c* value obtained by MRE of normal liver and FIB-4 in 51 HCC patients. **B**–**H** Comparisons of viscoelastic parameters related to laboratory indicators, imaging, and pathological characteristics of HCC. E–S, Edmondson–Steiner grade; APHE, arterial phase high enhancement; APHC, arterial phase hypovascular component; SN, satellite nodule; IA, intratumoral artery; IF, intratumoral fat; SM, smooth margin; FIB-4, fibrosis-4 score. “*” represents *p* < 0.05; “**” represents *p* < 0.01

**Fig. 3:**
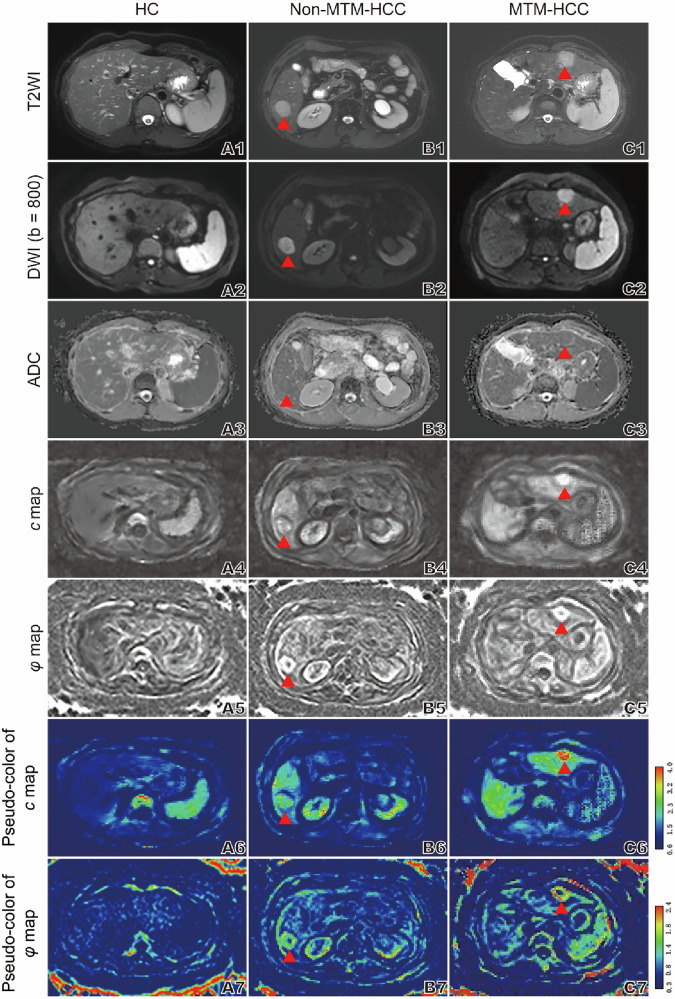
Representative imaging: Row 1: axial T2-weighted images; Row 2: axial diffusion-weighted images (DWI) at b value of 800 s/mm^2^; Row 3: corresponding apparent diffusion coefficient (ADC) maps; Row 4: axial MRE; Rows 5–7: *c* (stiffness) (Row 5) and *φ* (viscosity) (Row 6) maps and corresponding pseudo-color maps (Row 7) for three participants. **A1**–**A7** Images in a healthy control subject (HC). **B1**–**B7** Images in a participant with non-MTM-HCC in segment VI of liver (*c* = 1.918 m/s; *φ* = 1.345 radian). **C1**–**C7** Images in a participant with MTM-HCC in segment II of liver (*c* = 3.585 m/s; *φ* = 1.608 radian). The red triangles mark the HCC tumor

The interobserver agreement for the MR features and viscoelastic parameters was good to excellent (kappa and ICC values are in the range 0.730 to 1, see Supplementary Table S3). Among the 51 HCC patients, normal liver *c* value exhibited a strong correlation with the Fibrosis-4 score (FIB-4) (*r* = 0.658, *p* < 0.001) (Fig. 2A), and when 51 patients were grouped according to FIB-4 low or high threshold (< 1.45 or > 3.25), the difference in liver *c* value was statistically significant (Supplementary Fig. S2B, C), and alanine transaminase is weakly correlated with T-*φ* (Supplementary Fig. S2A). Compared with the HCCs of E–S I and II, the HCCs of E–S III and IV have higher T-*c* (2.55 ± 0.65 vs 2.18 ± 0.53; *p* = 0.027) (Fig. 2B), and the S-*c* value of HCC patients with satellite nodules was higher than that of HCC patients without satellite nodules (2.98 ± 0.49 vs 2.47 ± 0.48; *p* = 0.005) (Fig. 2E). HCCs with ≥ 20% APHC or Non-APHE signs on routine MRI have higher T-*c* values (respectively, 2.56 ± 0.65 vs 2.10 ± 0.47, *p* = 0.005 and 2.53 ± 0.61 vs 2.05 ± 0.50, *p* = 0.005) (Fig. 2C, D), and HCCs with Non-IF, Non-IA, or Non-SM signs may exhibit higher T-*φ* values (respectively, 1.15 ± 0.23 vs 0.92 ± 0.19, *p* = 0.016; 1.24 ± 0.28 vs 1.08 ± 0.20, *p* = 0.028; and 1.18 ± 0.20 vs 1.05 ± 0.25, *p* = 0.044) (Fig. 2F–H). Moreover, the viscoelastic parameters (*c* and *φ*) of tumors were significantly higher than those of normal liver tissue (*c*: 2.33 vs 1.71, *p* < 0.001; *φ*: 1.12 vs 0.82, *p* < 0.001) (Supplementary Fig. S1). The viscoelastic parameters of normal liver tissue in 51 HCC patients were also significantly elevated compared to those of the 47 healthy controls (*c*: 1.71 vs 1.37, *p* < 0.001; *φ*: 0.82 vs 0.69, *p* < 0.001) (Supplementary Fig. S1). Figure 3 demonstrates conventional MR images together with MRE-derived c and φ maps from healthy controls, non-MTM-HCC, and MTM-HCC patients.

### Diagnostic performance of viscoelastic values in detecting MTM-HCC

Based on pathological examination results as the gold standard, 51 patients were divided into the MTM-HCC group (*n* = 16) and the non-MTM-HCC group (*n* = 35). No statistically significant differences were observed in clinical blood biochemical parameters between the two groups (Table 1). Pathological examination revealed a higher prevalence of satellite nodules in MTM-HCC patients (7/16 [43.8%] vs 3/35 [8.6%]; *p* = 0.006) and a greater proportion of advanced E–S grades III and IV (11/16 [73.3%] vs 10/35 [28.6%]; *p* = 0.013) (Table 1). Patients with MTM-HCC exhibited significantly higher T-*c* (2.80 ± 0.58 vs 2.12 ± 0.50, *p* < 0.001) and T-*φ* (1.22 ± 0.23 vs. 1.07 ± 0.23, *p* = 0.028), and the S-*c* in patients with MTM-HCC was significantly elevated compared to patients with non-MTM-HCCs (2.79 ± 0.45 vs 2.47 ± 0.52, *p* = 0.039), while no statistically significant difference was observed in the *φ* of spleen tissue between the groups (Table 2). In addition, MTM-HCC patients were more likely to exhibit an APHC ≥ 20% (13/16 [81.3%] vs 13/35 [37.1%]; *p* = 0.009) (Table 3).

**Table 1:** Demographic and clinical characteristics of patients with and without MTM-HCC

Characteristic	MTM-HCC (*n* = 16)	Non-MTM-HCC (*n* = 35)	*p*-value
Sex, *n* (%)			
M	13 (81.3%)	29 (82.9%)	1.000
F	3 (18.8%)	6 (17.1%)	
Age > 50 years, *n* (%)	10 (62.5%)	30 (85.7%)	0.078
Underlying liver disease, *n* (%)			
HBV	13 (81.3%)	29 (82.9%)	0.172
HCV	0 (0%)	4 (11.4%)	
Other	3 (18.8%)	2 (5.7%)	
Neutrophil count (× 10^9^)†	3.2 (2.8–4.6)	3.6 (3.1–4.4)	0.759
Lymphocyte count (× 10^9^)†	1.2 (1.0–2.1)	1.6 (1.2–2.1)	0.282
Neutrophil/lymphocyte count†	2.6 (2.0–4.7)	2.1 (1.7–3.4)	0.270
Platelet count (× 10^9^)†	172.5 (129–214)	169 (137–214)	0.990
Albumin (g/L)†	43.8 (39.7–46.1)	41 (38.6–45.3)	0.131
Total bile acids (μmol/L)†	5.7 (3.3–12)	5.4 (3.3–13.2)	0.924
Creatine (μmol/L)†	65.7 (57.5–65.7)	74 (62–82)	0.693
Alanine aminotransferase (U/L)†	35.6 (22.7–55.9)	28.8 (23–39)	0.240
Aspartate aminotransferase (U/L)†	34.5 (31.3–37)	33 (25–42)	0.563
α-Fetoprotein, *n* (%)			
≤ 400 ng/mL	10 (62.5%)	29 (82.9%)	0.157
> 400 ng/mL	6 (37.5%)	6 (17.1%)	
Child–Pugh class, *n* (%)			
A	13 (81.3%)	33 (94.3%)	0.309
B	3 (18.8%)	2 (5.7%)	
C	0 (0%)	0 (0%)	
FIB-4 score, *n* (%)			
≤ 3.25	12 (75%)	26 (74.3%)	1.000
> 3.25	4 (25%)	9 (25.7%)	
ALBI grade, *n* (%)			
1	13 (81.3%)	22 (62.9%)	0.329
2	3 (18.8%)	13 (37.1%)	
3	0 (0%)	0 (0%)	
Satellite nodule, *n* (%)	7 (43.8%)	3 (8.6%)	0.006
Microvascular invasion, *n* (%)			
0	7 (43.8%)	25 (71.4%)	0.081
1	8 (50%)	7 (20%)	
2	1 (6.3%)	3 (8.6%)	
Edmondson–Steiner grade, *n* (%)			
I, II	5 (31.3%)	25 (71.4%)	0.013
III, IV	11 (68.8%)	10 (28.6%)	

Unless otherwise specified, data are numbers of patients, with percentages in parentheses

*FIB-4* fibrosis-4 score, *ALBI* albumin-bilirubin

† Data are medians with IQRs in parentheses

**Table 2:** Comparison of MRI features between MTM-HCC and non-MTM-HCC in 51 patients

Location	Characteristic	MTM-HCC (*n* = 16)	non-MTM-HCC (*n* = 35)	*p*-value
Tumor				
	*c* (m/s)	2.80 ± 0.58 (1.49–3.73)	2.12 ± 0.50 (1.25–3.23)	< 0.001
	*φ* (radian)	1.22 ± 0.23 (0.84–1.61)	1.07 ± 0.23 (0.69–1.57)	0.028
Normal liver				
	*c* (m/s)	1.65 ± 0.37 (1.25–2.36)	1.73 ± 0.33 (1.16–2.55)	0.472
	*φ* (radian)	0.79 ± 0.15 (0.48–1.03)	0.84 ± 0.19 (0.58–1.67)	0.692
Spleen				
	*c* (m/s)	2.79 ± 0.45 (2.23–3.69)	2.47 ± 0.52 (1.15–3.50)	0.039
	*φ* (radian)	0.93 ± 0.17 (0.56–1.24)	0.94 ± 0.14 (0.615–1.24)	0.924

Data are presented as mean ± standard deviation (range)

**Table 3:** Comparison of mechanical characteristics between macrotrabecular-massive hepatocellular carcinoma (MTM-HCC) and non-MTM-HCC in study participants

MRI feature	MTM-HCC (*n* = 16)	non-MTM-HCC (*n* = 35)	*p*-value
Main tumor size > 5 cm, *n* (%)	9 (56.3%)	20 (57.1%)	0.952
Lobulated/diffuse shape, *n* (%)	8 (50.0%)	17 (48.6%)	0.925
Non-smooth margin, *n* (%)	10 (62.5%)	16 (45.7%)	0.266
≥ 20% APHC, *n* (%)	13 (81.3%)	13 (37.1%)	0.009
Non-rim APHE, *n* (%)	3 (18.8%)	18 (51.4%)	0.058
Non-peripheral washout, *n* (%)	9 (56.3%)	25 (71.4%)	0.286
Enhancing capsule, *n* (%)	14 (87.5%)	32 (91.4%)	1.000
Non-enhancing capsule, *n* (%)	2 (12.5%)	1 (2.9%)	0.474
Nodule in nodule, *n* (%)	1 (6.8%)	8 (22.9%)	0.295
Mosaic architecture, *n* (%)	3 (18.8%)	7 (20.0%)	1.000
Intratumoral hemorrhage, *n* (%)	4 (25.0%)	10 (28.6%)	1.000
Intratumoral fat, *n* (%)	1 (6.3%)	6 (17.1%)	0.542
Peritumoral arterial enhancement, *n* (%)	4 (25.0%)	10 (28.6%)	1.000
Substantial intratumoral necrosis, *n* (%)	6 (37.5%)	12 (34.3%)	0.824
Intratumoral artery, *n* (%)	12 (75%)	26 (74.3%)	1.000
Delayed central enhancement, *n* (%)	2 (12.5%)	1 (2.9%)	0.474

Data are numbers of patients, with percentages in parentheses

*APHE* arterial phase high enhancement, *APHC* arterial phase hypovascular component

**Fig. 4:**
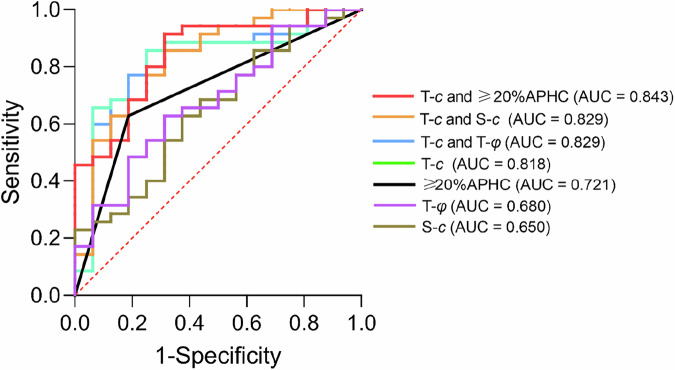
Receiver operating characteristic curves for assessing diagnostic utility regarding diagnostic differentiation of MTM-HCC. AUC, area under the operating characteristic curve (values are shown)

Multivariable logistic regression analysis showed that T-*c* was independently associated with the MTM subtype (odds ratio, 4.963; 95% confidence interval: 1.064–23.155; *p* = 0.041) (Table 4). The AUC for diagnosing MTM-HCC using T-*c* alone was 0.818 (95% confidence interval: 0.685–0.950; cutoff value: 2.462). When combining T-*c* and 20%APHC, the AUC was the highest (AUC = 0.843; 95% confidence interval: 0.729–0.957) (Fig. 4 and Table 5). However, no significant differences in AUC values were observed among these two models (Supplementary Table S4).

**Table 4:** Predictors for identifying MTM-HCC at logistic regression analysis in the training cohort

Variable	Univariable OR	*p*-value	Multivariable OR	*p*-value
≥ 20% APHC	7.333 (1.754, 30.657)	0.006	3.401 (0.554, 20.892)	0.186
Non-rim APHE	0.218 (0.053, 0.901)	0.035	0.998 (0.152, 6.555)	0.998
T-*c*	10.346 (2.459, 43.538)	0.001	4.963 (1.064, 23.155)	0.041
T-*φ*	21.102 (1.221, 364.59)	0.036	7.379 (0.197, 276.273)	0.280
S-*c*	4.046 (1.017, 16.099)	0.047	2.625 (0.528, 13.043)	0.238

Numbers in parentheses are 95% confidence intervals

*OR* odds ratio, *APHC* arterial phase hypovascular component, *APHE* arterial phase hyperenhancement, *T* tumor, *S* spleen

**Table 5:** Diagnostic performance of mechanical parameters in differentiation of hepatocellular carcinoma (MTM-HCC) and non-MTM-HCC

Parameter(s)	Cutoff value	AUC	Sensitivity	Specificity
T-*c*	2.462	0.818 (0.685, 0.950)	0.750	0.857
T-*φ*	1.131	0.680 (0.525, 0.836)	0.688	0.629
S-*c*	2.635	0.650 (0.489, 0.811)	0.625	0.629
≥ 20%APHC	……	0.721 (0.5723, 0.869)	0.813	0.629
T-*c* and ≥ 20%APHC	……	0.843 (0.729, 0.957)	0.688	0.914
T-*c* and S-*c*	……	0.829 (0.701, 0.956)	0.688	0.857
T-*c* and T-*φ*	……	0.829 (0.707, 0.950)	0.750	0.857

Area under the receiver operating characteristic curve (AUC) of each model is shown in the table. Data in parentheses are 95% confidence intervals

*T* tumor, *S* spleen, *APHC* arterial phase hypovascular component

### Transcriptomic differences between high-*c* and low-*c* groups’ hepatocellular carcinoma and associated biological pathways

**Fig. 5:**
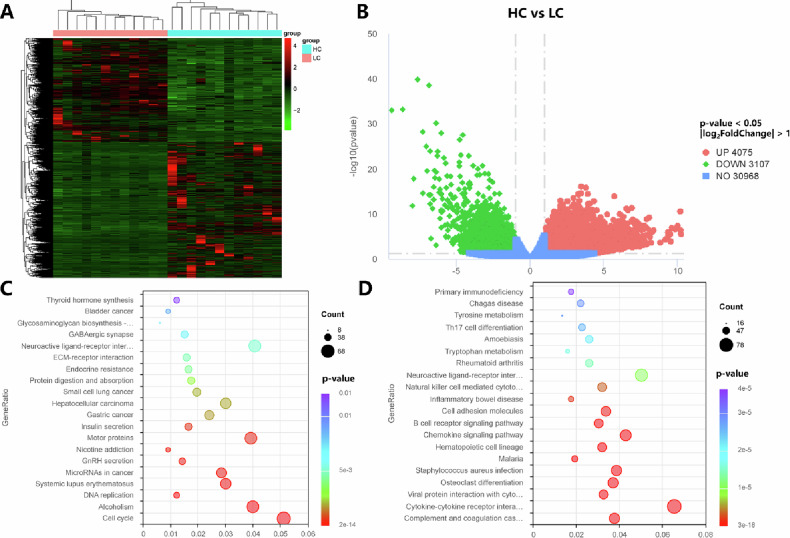
Differential gene expression and pathway enrichment analysis between high-stiffness and low-stiffness hepatocellular carcinoma samples. **A** Hierarchical clustering heatmap of differentially expressed genes between high-stiffness and low-stiffness groups. The heatmap shows the relative expression levels of genes, with red representing high expression and green representing low expression. **B** Volcano plot illustrating the distribution of differentially expressed genes between high- and low-stiffness groups. **C** KEGG pathway enrichment analysis of the top 500 upregulated genes in the high-stiffness group. **D** KEGG pathway enrichment analysis of the top 500 downregulated genes in the high-stiffness group. The size of the dots represents the number of genes enriched in each pathway, while the color scale reflects the *p*-value significance. HC, high stiffness; LC, low stiffness; UP, upregulated; DOWN, downregulated; NO, neither upregulated nor downregulated

**Fig. 6:**
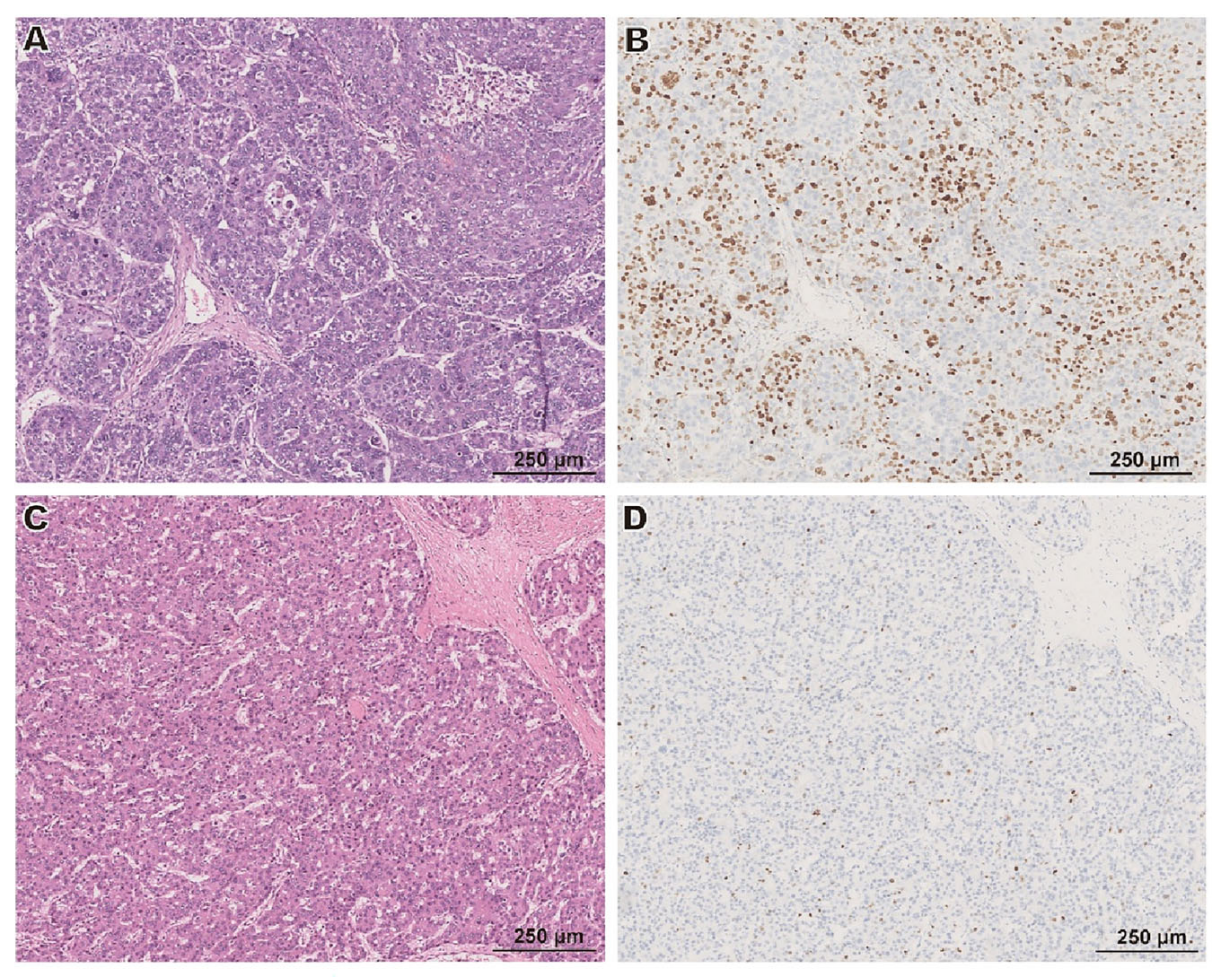
**A**, **B** HE staining (**A**) and Ki67 staining (**B**) of a specimen from a 42-year-old female patient in the high-stiffness group (with T-*c* value of 2.719 m/s); the specimen’s pathological classification was MTM-HCC. The Ki67 hotspot area accounted for approximately 60%. **C**, **D** HE staining (**C**) and Ki67 staining (**D**) of a specimen from a 58-year-old male patient in the low-stiffness group (with T-*c* value of 1.918 m/s); the specimen’s pathological classification was non-MTM-HCC, with the Ki67 hotspot area accounting for approximately 5%

Given the outstanding utility of the *c* value in identifying the aggressive subtype of MTM-HCC, we performed RNA sequencing analysis on 12 HCC samples, each from the high-*c* and low-c groups. The characteristics of High-c and Low-*c* patients are summarized in Supplementary Table S5, and the characteristics of patients in RNA sequencing samples are summarized in Supplementary Table S6. Initially, we conducted a clustering analysis between the two groups, which revealed significant differences in gene expression between high-*c* and low-*c* HCCs, while the homogeneity within each group remained stable. Further analysis of differentially expressed genes between the two groups, with the low-*c* group as the baseline, showed that 4075 genes had elevated expression in the high-*c* group, while 3107 genes had decreased expression. KEGG pathway enrichment analysis of the top 500 upregulated genes revealed enrichment in pathways related to the cell cycle, alcoholism, and DNA replication (Fig. 5), and pathological analysis revealed that tumors in the high-*c* group exhibited a higher Ki67 positivity rate (Fig. 6). KEGG pathway enrichment analysis of the top 500 downregulated genes showed these genes were enriched in pathways, such as complement and coagulation cascades and cytokine–cytokine receptor interactions (Fig. 5).

## Discussion

MRE is a promising noninvasive imaging technique that enables the characterization of mechanical properties, which have been increasingly linked to cancer aggressiveness. However, to our knowledge, studies characterizing the biomechanical properties (including stiffness and viscosity) of the aggressive MTM-HCC subtype remain limited. In this study, tumor stiffness (T-*c* value) served as a noninvasive marker for MTM-HCC, yielding an ROC of 0.818. Furthermore, RNA sequencing identified differential gene expression in HCC tumors across distinct stiffness levels.

Previous studies have demonstrated that MRE-derived HCC stiffness not only correlates with pathological grade, microvascular invasion, vessels encapsulating tumor clusters, and Ki67/CK19 levels, but also provides valuable patient prognostic information. [10, 11, 25, 26]. The results of our study also show that HCCs with higher E–S grades (III and IV), satellite nodules, non-smooth tumor margins, fat deficiency, or ≥ 20%APHC features are more likely to have higher tumor stiffness (*c*) or viscosity (*φ*). Tumor stiffness may serve as a critical determinant influencing MR enhancement characteristics in the arterial phase, while the viscosity of tumor tissue might be associated with intratumoral blood supply. Hypovascular HCCs are prone to hypoxic necrosis, resulting in viscosity within the tumor. Additionally, our findings reveal that patients with fat-positive tumors have lower *φ* values, and related studies suggest that HCC patients with intratumoral fat tend to have a better prognosis [27]. These findings collectively suggest that higher viscoelastic values of HCC demonstrate significant correlations with increased invasiveness and unfavorable clinical outcomes.

In this study, we investigated the stiffness and viscosity of MTM-HCC, an aggressive hepatocellular carcinoma subtype, and we observed that MTM-HCC exhibited significantly higher T-*c* and T-*φ*, suggesting changes at both the molecular and physicochemical levels in MTM-HCC. Increased stiffness indicates more severe fibrous hyperplasia [28], which may be the cause of insufficient blood supply in the arterial phase of the mass and lead to hypoenhancement in the arterial phase [21]. Tumors with high viscosity tend to have increased potential for early recurrence and metastasis, as they may facilitate the movement of cancer cells through the extracellular matrix and surrounding tissues [29]. The high stiffness and high viscosity of tumors further support the notion that MTM-HCCs are more aggressive. Furthermore, our study contributes to the growing body of evidence highlighting the importance of spleen stiffness in predicting the recurrence of HCC. The results indicate that the spleen stiffness value of patients with MTM-HCC is higher than that of patients with non-MTM-HCC, which aligns with previous research linking spleen stiffness to portal hypertension and HCC prognosis [30, 31]. To assess the role of viscoelastic parameters and MRI features in identifying the MTM subtype, we conducted univariate and multivariate analyses, which revealed that both parameters were associated with the MTM subtype. Through ROC curve analysis, we found that the combination of T-*c* and ≥ 20%APHC demonstrated the best diagnostic performance for MTM-HCC (AUC = 0.846). This suggests that integrating tumor physical properties with conventional MRI features may provide a better approach for clinical assessment and analysis. However, in this study, the diagnostic performance of the T-*c* value alone was relatively high (AUC = 0.818), indicating that the increased tumor stiffness may be a key characteristic in MTM-HCC.

Considering these factors, we conducted RNA sequencing analysis to explore gene expression differences across various stiffness levels. Our findings show that genes highly expressed in high-*c* HCCs are primarily associated with tumor proliferation pathways, whereas genes with lower expression in high-*c* HCCs are linked to immune-related pathways. The MTM-HCC subtype is characterized by high proliferative activity and an immune desert phenotype [32], and these partial findings may provide an explanation for the predictive value of tumor stiffness in identifying the MTM-HCC subtype. Additionally, these findings suggest that tumor stiffness may provide evidence to support immunotherapy and predict patient prognosis, but further studies are required to validate the clinical relevance of tumor stiffness.

The study has several limitations. First, the cohort was relatively small and predominantly comprised HBV-associated HCC, with a higher MTM-HCC subtype prevalence (< 20%) than previously reported, potentially limiting generalizability. Future validation in larger cohorts, particularly Western populations with metabolic dysfunction-associated steatotic liver-disease-driven HCC, is warranted. Second, despite careful variable selection, the small sample size and lack of external validation increase overfitting risk and compromise robustness, necessitating multicenter validation. Third, inclusion of only surgical patients with preserved liver function may introduce selection bias; future studies should include advanced HCC cases. Finally, RNA sequencing involved a limited sample size and requires functional validation. In summary, while our exploratory findings show potential clinical relevance, robust validation in larger, heterogeneous multicenter cohorts is essential to confirm clinical utility.

In conclusion, our research indicates tumor stiffness quantified by the viscoelastic parameter T-*c* effectively distinguishes MTM-HCC from other subtypes. Moreover, differential gene expression associated with distinct tumor stiffness levels was identified. These findings support the role of MRE-derived tumor stiffness as a noninvasive imaging biomarker for identifying MTM-HCC, with potential to guide personalized treatment. Validation in larger, diverse cohorts is necessary to establish its clinical value.

## Supplementary information

ELECTRONIC SUPPLEMENTARY MATERIAL

## Abbreviations

APHC: Arterial phase hypovascular component
APHE: Arterial phase hyperenhancement
AUC: Area-under-the-curve
DWI: Diffusion-weighted images
E-S grade: Edmondson-Steiner grade
FIB-4: Fibrosis-4
HBV: Hepatitis B virus
HC: Healthy control
HCC: Hepatocellular carcinoma
HCV: Hepatitis C virus
HE staining: Hematoxylin-eosin staining
IA: Intratumoral artery
ICC: Intraclass correlation coefficient
IF: Intratumoral fat
KEGG: Kyoto Encyclopedia of Genes and Genomes
MRE: Magnetic-resonance elastography
MTM: Macrotrabecular-massive
Non-SM: Non-smooth margin
ROC: Receiver operating characteristic
ROI: Regions of interest
S-*c*: Spleen-*c*
SM: Smooth margin
T2WI: T2-weighted image
T-*c*: Tumor-*c*
T-*φ*: Tumor-*φ*
