# Supplementary material for: Detection of macrotrabecular-massive hepatocellular carcinoma based on viscoelastic characteristics obtained by multifrequency magnetic-resonance elastography
Source: Eur Radiol. 2025 Oct 9;36(4):2388–400. doi: 10.1007/s00330-025-12024-y (PMC13035537; doi:10.1007/s00330-025-12024-y)
Supplement: Supplementary file 1 — ELECTRONIC SUPPLEMENTARY MATERIAL [file 330_2025_12024_MOESM1_ESM.docx]

**Table S1** Conventional MRI features and their definitions

| **MRI features** | **Definition** |
| --- | --- |
| 1. Margin of tumor | Non-smooth or smooth |
| 2. Shape of tumor | Tumor with a round/oval shape or lobulated/diffuse shape |
| 3. Tumor size ≥ 5cm | The observed maximum section of the outer edge - outer edge diameter≥5 cm |
| 4. APHC ≥ 20% | Arterial phase hypovascular component ≥ 20% |
| 5. Non-rim APHE | Tumor enhancement at the arterial phase that is unequivocally greater in whole or in part than the surrounding liver |
| 6. Nonperipheral washout | The signal of the mass in the portal or delayed phase is reduced compared to the surrounding liver |
| 7. Enhancing capsule | Tumors with an enhancing capsule observed at the portal venous phase or the delayed phase |
| 8. Non-enhanced capsule | The capsule does not enhance in portal phase or delayed phase, showing low signal |
| 9.Nodule-in-nodule | Partial nodular hyperenhancement within a hypovascular nodule at the arterial phase |
| 10. Mosaic architecture | Randomly distributed internal nodules or compartments with variable imaging features and separated by septations |
| 11. Intratumoral hemorrhage | A hyperattenuated area with lower attenuation than calcification on the nonenhanced images |
| 12. Intratumoral fat | The overall or local signal in the out-phase tumor is reduced compared with that of the in-phase |
| 13. Arterial phase peritumoral enhancement | A fuzzy-marginated hyperenhancement portion at the arterial phase adjacent to the tumor and becoming isointense compared with the background liver parenchyma at the portal venous phase or delayed phase |
| 14. Substantial Intra-tumoral necrosis | A hypointense central area on unenhanced phase, without enhancement on postcontrast phases involving more than 20% of the tumor area on the largest cross-sectional images |
| 15. Intratumoral artery | The persistence of discrete arteries within the tumor |
| 16. Central enhancement at delayed phase | Peripheral enhancement with progressive central filling |
| 17. Ascites | Small, medium, and large amounts of ascites |

**Table S2**  Demographic and clinical characteristics of non-MTM-HCC subtypes and healthy control

| **Characteristic** | **Total HCC**  **(n = 51)** | **Trabecular (n = 24)** | **Pseudo-glandular**  **(n = 6)** | **Solid**  **(n = 5)** | **Healthy control**  **(n = 47)** |
| --- | --- | --- | --- | --- | --- |
| Sex *n* (%) |  |  |  |  |  |
| M | 42 (82.4%) | 21 (87.5%) | 4(66.7%) | 4(80.0%) | 24 (51.1) |
| F | 9 (17.6%) | 3 (12.5%) | 2 (33.3%) | 1 (20.0%) | 23 (48.9) |
| Age years±SD (range) | 56.2±12.6 (30–77) | 59.6±11.3（31–72） | 54.7±12.6（32–69） | 59.6±17.0（33–77） | 54.1±13.7 (25–77) |
| Liver disease *n* (%) |  |  |  |  |  |
| HBV | 42 (82.4%) | 21 (87.5%) | 4 (66.7%) | 4 (80%) | …… |
| HCV | 4 (7.8%) | 2 (8.3%) | 1 (16.7%) | 1 (20%) | …… |
| Other | 5 (9.8%) | 1 (4.2%) | 1 (16.7%) | 0 (0%) | …… |
| NEUT (⨯10^9^ )† | 3.6 (3.0–4.4) | 3.6 (3.1–4.1) | 4.5 (2.2–5.7) | 3.1 (2.4–4.0) | …… |
| LYMPH (⨯10^9^ )† | 1.6 (1.1–2.1) | 1.6 (1.2–2.3) | 1.2 (0.6–1.5) | 1.6 (1.4–1.9) | …… |
| NEUT/LYMPH† | 2.3 (1.7–3.5) | 2.0 (1.6–3.2) | 3.8 (3.2–4.7) | 1.7 (1.5–2.5) | …… |
| PLT (⨯10^9^ )† | 169 (130–214) | 167(132–211) | 175 (84–254) | 189 (168–218) | …… |
| ALB (g/L)† | 42.4 (38.8–45.4) | 41.4 (38.7–45.3) | 41.4 (38.7–45.6) | 38 (36.5–45.6) | …… |
| TBA (μmol/L)† | 5.4 (3.3–12) | 4.5 (3.3–16.3) | 8 (3.9–11.9) | 5.4 (2.6–15.2) | …… |
| Cr (μmol/L)† | 72 (62–81) | 76.2 (62.3–82) | 61.5 (45.3–81) | 68 (55.5–81) | …… |
| ALT (U/L)† | 30 (23–47) | 30.7 (24.4–53.3) | 25 (18.5–33.3) | 25 (18.5–34) | …… |
| AST (U/L)† | 33.7 (26–41) | 33.4 (25.3–44.4) | 35 (23.8–71.5) | 26 (23–33) | …… |
| AFP *n* (%) |  |  |  |  |  |
| ≤400 ng/mL | 39 (76.5%) | 22 (91.7%) | 3 (50%) | 4 (80%) | …… |
| ＞400 ng/mL | 12 (23.5%) | 2 (8.3%) | 3 (50%) | 1 (20%) | …… |
| C–P class *n* (%) |  |  |  |  |  |
| A | 46 (90.2%) | 23 (95.8%) | 6 (100%) | 4 (80%) | …… |
| B | 5 (9.8%) | 1 (4.2%) | 0 (0%) | 1 (20%) | …… |
| C | 0 (0%) | 0 (0%) | 0 (0%) | 0 (0%) | …… |
| FIB-4 score *n* (%) |  |  |  |  |  |
| ≤ 3.25 | 38 (74.5%) | 17 (70.8%) | 5 (83.3%) | 4 (80%) | …… |
| ＞ 3.25 | 13 (25.5%) | 7 (29.2%) | 1 (16.7%) | 1 (20%) | …… |
| ALBI grade *n* (%) |  |  |  |  |  |
| 1 | 35 (68.9%) | 15 (62.5%) | 4 (66.7%) | 3 (60%) | …… |
| 2 | 16 (31.4%) | 9 (37.5%) | 2 (33.3%) | 2 (40%) | …… |
| 3 | 0 (0%) | 0 (0%) | 0 (0%) | 0 (0%) | …… |
| SN *n* (%) | 10 (19.6%) | 2 (8.3%) | 1(16.7%) | 1 (16.7%) | …… |
| MVI *n* (%) |  |  |  |  |  |
| 0 | 32 (62.7%) | 16 (66.7%) | 5 (83.3%) | 4 (80%) | …… |
| 1 | 15 (29.4%) | 5 (20.8%) | 1 (16.7%) | 1 (20%) | …… |
| 2 | 4 (7.8%) | 3 (12.5%) | 0 (0%) | 0 (0%) | …… |
| E–S grade *n* (%) |  |  |  |  |  |
| I, II | 30 (58.8) | 19 (79.2%) | 4 (66.7%) | 2 (40%) | …… |
| III, IV | 21 (41.2) | 5 (20.8%) | 2 (33.3%) | 3 (60%) | …… |

Unless otherwise specified, data presented as number of patients, with percentages in parentheses. NEUT, Neutrophil; LYMPH, Lymphocyte; PLT, Platelet; ALB, Albumin; TBA, Total bile acids; Cr, Creatine; ALT, Alanine aminotransferase; AST, Aspartate aminotransferase; AFP, α-Fetoprotein; C-P class, Child–Pugh class; FIB-4, Fibrosis 4 Score; ALBI, Albumin-Bilirubin; SN, Satellite nodule; MVI, Microvascular invasion; E-S grade, Edmondson–Steiner grade.

†Data are medians with IQRs in parentheses.

**Table S3** Intraclass correlation coefficient and κ coefficient

| Features | Kappa/ICC value | *p* |
| --- | --- | --- |
| Margin of tumor † | 0.921 | ﹤0.001 |
| Shape of tumor † | 0.804 | ﹤0.001 |
| Tumor size ≥ 5cm † | 1.000 | ﹤0.001 |
| APHC ≥ 20% † | 0.882 | ﹤0.001 |
| Non-rim APHE † | 0.799 | ﹤0.001 |
| Non-peripheral washout † | 0.743 | ﹤0.001 |
| Enhancing capsule † | 0.778 | ﹤0.001 |
| Non-enhanced capsule † | 0.847 | ﹤0.001 |
| Nodule-in-nodule † | 0.852 | ﹤0.001 |
| Mosaic architecture † | 0.751 | ﹤0.001 |
| Intratumoral hemorrhage | 0.897 | ﹤0.001 |
| Intratumoral fat † | 0.766 | ﹤0.001 |
| Peritumoral arterial enhancement † | 0.736 | ﹤0.001 |
| Delayed central enhancement † | 0.79 | ﹤0.001 |
| Substantial Intratumoral necrosis † | 0.783 | ﹤0.001 |
| Intratumoral artery † | 0.897 | ﹤0.001 |
| Ascites † | 1.000 | ﹤0.001 |
| T-*c* ‡ | 0.891 | ﹤0.001 |
| T-*φ* ‡ | 0.878 | ﹤0.001 |
| P-*c* ‡ | 0.869 | ﹤0.001 |
| P-*φ* ‡ | 0.730 | ﹤0.001 |
| S-*c* ‡ | 0.918 | ﹤0.001 |
| S-*φ* ‡ | 0.761 | ﹤0.001 |

APHC**,** arterial phase hypovascular component; APHE, arterial phase hyperenhancement; T, tumor; P, paratumoral; S, spleen, c, stiffness; *φ*, viscosity. †: Cohen’s kappa for categorical variable; ‡: intraclass correlation coefficient for continuous variable

**Table S4** DeLong test compares AUC differences among models

| **Model comparison** | **AUC difference** | ***p*-value** |
| --- | --- | --- |
| T-*c* vs T-*φ* | 0.137 | 0.179 |
| T-*c* vs ≥20%APHC | 0.097 | 0.261 |
| T-*c* vs S-c | 0.168 | 0.065 |
| T-*c* vs T-*c* and ≥20%APHC | -0.025 | 0.533 |
| T-c vs T-*c* and T-*φ* | -0.011 | 0.625 |
| T-*c* vs T-*c* and S-*c* | -0.011 | 0.674 |
| T-φ vs ≥20%APHC | -0.040 | 0.691 |
| T-φ vs S-*c* | 0.030 | 0.810 |
| T-φ vs T-*c* and 20%APHC | -0.162 | 0.087 |
| T-φ vs T-*c* and T-*φ* | -0.148 | 0.093 |
| T-φ vs T-*c* and S-*c* | -0.148 | 0.147 |
| ≥20%APHC vs S-*c* | 0.071 | 0.459 |
| ≥20%APHC vs T-*c* and ≥20%APHC | -0.122 | **0.016** |
| ≥20%APHC vs T-*c* and T-*φ* | -0.108 | 0.192 |
| ≥20%APHC vs T-*c* and S-*c* | -0.108 | 0.172 |
| S-c vs T-*c* and ≥20%APHC | -0.193 | **0.019** |
| S-c vs T-*c* and T-*φ* | -0.179 | **0.050** |
| S-c vs T-*c* and S-*c* | -0.179 | **0.012** |
| T-*c*+≥20%APHC vs T-*c* and T-*φ* | 0.014 | 0.725 |
| T-*c* and ≥20%APHC vs T-*c* and S-*c* | 0.014 | 0.692 |
| T-*c* and T-*φ* vs T-*c* and S-*c* | 0.000 | 1.000 |

Seven models (T-*c*, T-*φ*, S-*c*, ≥20%APHE, T-*c* and ≥20%APHC, T-*c* and S-*c*, and T-*c* and T-*φ*) were used to diagnose MTM-HCC. The lowest *p*-values achieved are set in boldface type.

**Table S5** Demographic and clinical characteristics of high-c and low-c patients

| **Characteristic** | **High-c**  **(n = 16)** | **Low-c**  **(n = 35)** |
| --- | --- | --- |
| Sex *n* (%) |  |  |
| M | 12 (75%) | 30 (85.7%) |
| F | 4 (25%) | 5 (14.3%) |
| Age years±SD (range) | 50.9±12.1(33–72) | 58.6±12.2(30–77) |
| Liver disease *n* (%) |  |  |
| HBV | 14 (87.5%) | 28 (80%) |
| HCV | 1 (6.3%) | 3 (8.6%) |
| Other | 1 (6.3%) | 4 (11.4%) |
| NEUT (⨯10^9^ )† | 3.4 (3.0–4.1) | 3.6 (3.0–4.6) |
| LYMPH (⨯10^9^ )† | 1.7 (1.0–2.3) | 1.5 (1.1–1.9) |
| NEUT/LYMPH† | 2.1 (1.8–3.1) | 3.4 (1.7–3.8) |
| PLT (⨯10^9^ )† | 183.5 (142.5–214) | 167 (126–214) |
| ALB (g/L)† | 42.7 (38.7–46.1) | 42.4 (38.8–45.2) |
| TBA (μmol/L)† | 4.3 (2.7–10) | 7.3 (3.9–17.3) |
| Cr (μmol/L)† | 69.5 (63–79) | 74 (61–82) |
| ALT (U/L)† | 37.5 (24.8–56.4) | 28.8(22-39) |
| AST (U/L)† | 33.8(25-37) | 33.7 (26–43) |
| AFP *n* (%) |  |  |
| ≤400 ng/mL | 9 (56.3%) | 30 (85.7%) |
| ＞400 ng/mL | 7 (43.8%) | 5 (14.3%) |
| C–P class *n* (%) |  |  |
| A | 14 (87.5%) | 32 (91.4%) |
| B | 2 (12.5%) | 3 (8.6%) |
| C | 0 (0%) | 0 (0%) |
| FIB-4 score *n* (%) |  |  |
| ≤ 3.25 | 14 (87.6%) | 24 (68.6%) |
| ＞ 3.25 | 2 (12.4%) | 11 (31.4%) |
| ALBI grade *n* (%) |  |  |
| 1 | 12 (75%) | 23 (65.7%) |
| 2 | 4 (25%) | 12 (34.3%) |
| 3 | 0 (0%) | 0 (0%) |
| SN *n* (%) | 6 (37.5%) | 4 (11.4%) |
| MVI *n* (%) |  |  |
| 0 | 7 (43.8%) | 25 (71.4%) |
| 1 | 8 (50%) | 7 (20%) |
| 2 | 1(6.2%) | 3(8.6%) |
| E–S grade *n* (%) |  |  |
| I, II | 6 (37.5%) | 24 (68.6%) |
| III, IV | 10 (62.5%) | 11 (31.4%) |
| Subtype *n* (%) |  |  |
| Macrotrabecular-massive | 11 (68.8%) | 5 (14.3%) |
| Trabecular | 4 (25.0%) | 20 (57.1%) |
| Pseudoglandular | 0 (0%) | 6 (17.1%) |
| Solid | 1 (6.3%) | 4 (11.4%) |

ALT, Alanine aminotransferase; AST, Aspartate aminotransferase; TBA, Total bile acids; NEUT, Neutrophil count; LYMPH, Lymphocyte; PLT, Platelet count; ALB, Albumin; Cr, Creatine; AFP, α-Fetoprotein; C-P class, Child–Pugh class; SN, Satellite nodule; MVI, Microvascular invasion; E-S grade, Edmondson–Steiner grade).

**Table S6** Clinical and pathologic characteristics of patients in RNA-sequencing samples

| **Characteristic** | **High-c**  **(n = 12)** | **Low-c**  **(n =12)** |
| --- | --- | --- |
| Sex *n* (%) |  |  |
| M | 8 (66.7%) | 10 (83.3%) |
| F | 4 (33.3%) | 2 (16.7%) |
| Age years±SD (range) | 51.6±12.2 (33–72) | 60.2±13.2 (30–77) |
| Liver disease *n* (%) |  |  |
| HBV | 11 (91.7%) | 10 (83.3%) |
| HCV | 1 (8.3%) | 0 (0%) |
| Other | 0 (0%) | 2 (16.7%) |
| Subtype *n* (%) |  |  |
| Macrotrabecular-massive | 9 (75%) | 2 (16.7%) |
| Trabecular | 2 (16.7%) | 8 (66.7%) |
| Pseudoglandular | 0 (0%) | 1 (8.3%) |
| Solid | 1 (8.3%) | 1 (8.3%) |


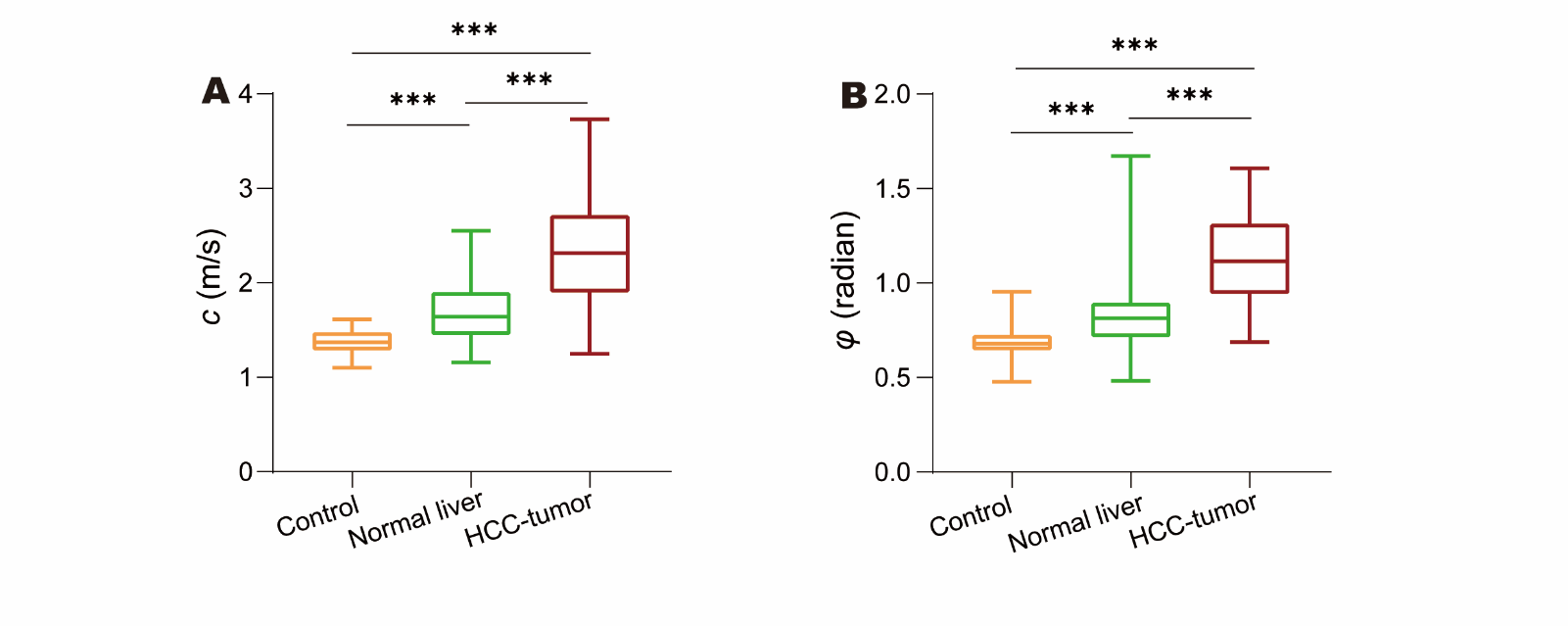


**Fig. S1** **A**, **B** Comparison of *c* value (**A**) and *φ* value (**B**) of liver and tumor in healthy control group and HCC patients. “***” represents *p* < 0.001.


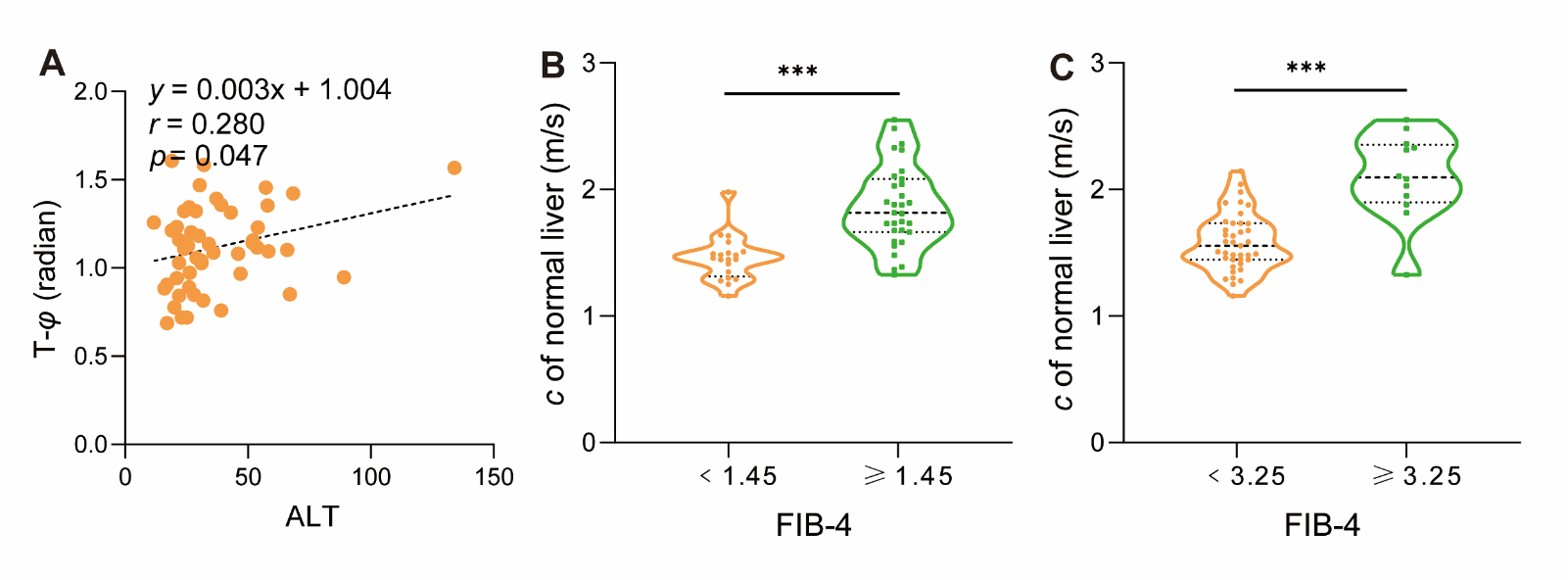


**Fig. S2** **A** The correlation between T-*φ* and ALT; **B**, **C** Group comparison of *c* values with two FIB-4 cutoff values of 1.45 (**B**) and 3.25 (**C**). ALT, alanine transaminase; FIB-4, Fibrosis-4 score. “***” represents *p* < 0.001.

**Magnetic resonance scan sequence**

All participants fasted for at least 4 hours prior to the examination and were positioned supine during the procedure.

**1. T1-FFE（fast field echo）(Axi/Cor)**

TR：150–200 ms ; TE: double echo in phase (≈ 2.3 ms) and reverse phase (≈ 1.15 ms);

Turning agle (FA): 70°–80°; Layer thickness: 5 mm, layer spacing: 1 mm; matrix: 256 ⨯ 192; FOV: 350 ⨯ 350 mm (adjusted according to body type); Scanning time: hold breath for 15–20 s.

**2. T2-SPAIR (fat inhibition) (Axi/Cor)**

TR: 2000–3000 ms (adjusted according to respiratory rate in respiratory trigger mode); TE: 80–100 ms (best liver parenchyma display); Turning angle: 90°; Layer thickness: 5 mm, layer spacing: 1 mm; matrix: 320 ⨯ 256; FOV: 350 ⨯ 350 mm; Fat inhibition: SPAIR; Parallel acquisition: SENSE acceleration factor 2–3; Scan time: 2–4 min (breathing trigger),

**3. In/out phase imaging (Axi)**

TR: 150–200 ms; TE1 (inverse phase); 1.15 ms (3.0 T water and fat signal cancellation); TE2 (in phase): 2.3 ms; Turning Angle: 70°; Layer thickness/distance: consistent with the T1WI; matrix: 256×192; Scanning time: hold breath for 15–20 s.

**4. Diffusion weighted imaging (DWI-SE–EPI) (axis)**

b value: 0, 50, 800 s/mm² (or clinical use of 0, 500, 1000); TR: 2000–4000 ms (breathing triggered or free breathing);TE: Minimum effective value (≈ 50–70 ms, system automatic optimization); Layer thickness: 5 mm, layer spacing: 1 mm; matrix: 128 ×128 (improved SNR); FOV: 350 x 350 mm;Fat inhibition: SPIR or STIR (reduce chemical shift artifacts); Parallel acquisition: SENSE acceleration factor 2–3; Scanning time: 2–3 minutes.

**5. 3D-elastography (RL-3**×**3**×**5-noGMN)**

Mechanical vibrations at 30, 40, 50, and 60 Hz were generated by four surface pressure pads powered by an air compressor, propagating vibrational waves into the liver. The two anterior and two posterior pads, centered over the liver region, operated at pressures of 0.4 bar and 0.6 bar, respectively. A small sandbag was placed above the anterior pads to enhance the transmission of vibrational waves. Three-dimensional wave fields were acquired using a single-shot spin-echo echo-planar imaging sequence, capturing 25 contiguous slices during free breathing, with a total acquisition time of 3 min and 56 s.

Scanning plane: axis; Parameter: FOV: 334 × 415; Fat suppression: SPAIR; Layer thickness: 5 mm; TR: 1200; TE: 36; matrix: 112 × 139; Turn angle: 90°; Scanning time: 3 min 56 s ; free breathing.

**6. Dynamic enhanced scanning (DCE)**

TR: 3.5–4.5 ms; TE: 1.5.0 ms; Turning Angle: 12°–15°; Layer thickness: 3–4 mm (no spacing); Matrix: 256 × 256; Fat suppression: SPAIR.

Scanning phase:Early arterial stage: 20–25 seconds after contrast injection (trigger scan); Advanced arterial stage: 30–35 seconds; Portal phase: 60–70 seconds; Delay period: 3 min (routine), 20 min (hepatobiliary specific contrast agents, such as Primexien); Scanning time: 15–20 s per session (complete with breath holding)

Hepatobiliary specific scan (optional):TR/TE: The same dynamic enhancement sequence; Delay time: 20 min after injection of contrast agent (Pumexian); Layer thickness: 3–4 mm.

**Enhanced scanning contrast agent solution:**

Contrast agent: Disodium gadoselate (Primecin) or gadolinium-diethylenetriaminepentaacetic acid

Dosage: 0.1 mmol/kg (or according to the instructions); Injection rate: 1.5–2 mL/s (normal saline flushing)
